# Implications of BCRP modulation on PTZ-induced seizures in mice: Role of ko143 and metformin as adjuvants to lamotrigine

**DOI:** 10.1007/s00210-023-02485-7

**Published:** 2023-04-17

**Authors:** Sahar A. Harby, Nehal A. Khalil, Norhan S. El-Sayed, Eman H. Thabet, Samar R. Saleh, Mona Hassan Fathelbab

**Affiliations:** 1 Department of Clinical Pharmacology, Faculty of Medicine, Alexandria University, Alexandria, Egypt; 2 Department of Medical Biochemistry, Faculty of Medicine, Alexandria University, Alexandria, Egypt; 3 Department of Medical Physiology, Faculty of Medicine, University of Alexandria, Alexandria, Egypt; 4 Center of Excellence for Research in Regenerative Medicine and Its Application (CERRMA), Faculty of Medicine, University of Alexandria, Alexandria, Egypt; 5 Department of Biochemistry, Faculty of Science, Alexandria University, Alexandria, Egypt; 6 Bioscreening and Preclinical Trial Lab, Department of Biochemistry, Faculty of Science, Alexandria University, Alexandria, Egypt

**Keywords:** Blood–brain barrier, BCRP, Epilepsy, ko143, Metformin, PTZ

## Abstract

Blood–brain barrier (BBB) efflux transporters' overexpression hinders antiepileptic drug brain entry. Breast cancer resistance protein (BCRP) is a major BBB efflux transporter. In the present work, BCRP's role as a mechanism that might contribute to drug-resistant epilepsy (DRE) in a mouse model of acute seizures was studied with further assessment of the effect of its inhibition by ko143 and metformin (MET) on lamotrigine (LTG) bioavailability and efficacy. 42 male mice divided into 6 groups: G1: Normal control, G2: LTG-injected healthy mice: LTG 20 mg/kg i.p., G3: Acute seizures (A.S) mice: Pentylenetetrazole (PTZ) 50 mg/kg i.p., G4: LTG-treated A.S mice: LTG 20 mg/kg + PTZ 50 mg/kg i.p., G5: Ko143 + LTG treated A.S mice: Ko143 15 mg/kg i.p. before LTG + PTZ, G6: MET + LTG treated A.S mice: MET 200 mg/kg i.p. before LTG + PTZ. Seizures severity, serum, brain LTG, and brain BCRP were assessed. PTZ group experienced the highest seizure frequency and brain BCRP expression. Ko143 and MET groups showed a significant decrease in brain BCRP with subsequent improvement in brain LTG level and better seizure control. BCRP has a significant role in epilepsy resistance and its inhibition with ko143 or MET adds value to DRE management.

**Supplementary Information:**

The online version contains supplementary material available at 10.1007/s00210-023-02485-7.

## Introduction

Despite a better understanding of epilepsy pathogenesis and the development of a wide range of antiepileptic drugs (AEDs), still, one-third of epilepsy patients do not respond to currently available therapies. This condition is referred to as drug-resistant epilepsy (DRE) (Lalitha et al. 2018). DRE imposes a threat to patients’ lives by increasing risks of injuries, psychosocial dysfunction, and even sudden death (Löscher et al. 2020; Shlobin and Sander 2022).

Consequently, the development of more effective AEDs is a critical therapeutic need. However, this issue is further complicated by the diversity of epilepsy types, as well as the puzzle of refractoriness which expands to include several hypotheses (Löscher et al. 2020), of which, the transporter hypothesis has received recently special attention. (Löscher and Friedman 2020; Czornyj et al. 2022).

The transporter hypothesis refers to multidrug resistance by efflux transporters overexpression at the blood–brain barrier (BBB) (Löscher and Friedman 2020). Most AEDs passively diffuse through BBB. However, the presence of protective transporters such as P-glycoprotein (P-gp; ABCB1) at BBB actively extrudes AED back into the blood and hinders their passage to their targets (Baltes et al. 2007). This delineates an additional pathophysiological mechanism of DRE where efflux transporters are overexpressed at BBB in specific epileptic brain foci (Löscher and Friedman 2020).

Studies on chemotherapy-resistant cancer were the first to demonstrate this theory (Amawi et al. 2019). Thereafter, it has gained much attention as a possible mechanism to explain resistance to several AEDs, regardless of their mechanism of action (Tang et al. 2017). Overexpressed P-gp has been detected in resected brain tissues in both experimental and clinical DRE studies (Pekcec et al. 2009; Koubeissi 2013). Another important efflux transporter that plays a significant role in drug disposition is the breast cancer-resistant protein (BCRP) (Ghosh et al. 2013; Banerjee Dixit et al. 2017).

Breast cancer resistance protein (BCRP; ABCG2), is considered one of the major BBB efflux transporters, where it controls brain diffusion of many lipophilic xenobiotics for brain protection (König et al. 2013). Despite the evidence of its potential role in restricting several drugs' brain entry (Mahringer and Fricker 2016; Saunders et al. 2016), reports related to its contribution to DRE are still scarce (Sisodiya et al. 2003; van Vliet et al. 2005).

Lamotrigine (LTG) is an AED approved for treating primary generalized tonic–clonic epilepsy and Lennox-Gastaut syndrome (Betchel et al. 2022).

Interestingly, LTG has been identified as a BCRP substrate. Since then, it is considered the optimal AED to study BCRP's role in epilepsy and the impact of its inhibition in overcoming DRE (Römermann et al. 2015).

Numerous compounds have been thoroughly investigated for their role in BCRP inhibition (Ahmed-Belkacem et al. 2006; Ni et al. 2010). Among these inhibitors, is the safe tetracyclic analogue of the fungal toxin fumitremorgin C (FTC); Ko143 which is distinguished by being a highly potent and selective BCRP inhibitor (Ni et al. 2010; Lustig et al. 2022). Furthermore, several other medications have been repurposed and have been proven to be transporter inhibitors as well, for example, metformin (MET) (Liang et al. 2015).

The pharmacokinetic studies of Metformin (MET), a first-line therapy for type 2 diabetes mellitus, revealed that it is a BCRP substrate with BBB permeability (Gong et al. 2012; Moreira 2014; Liang et al. 2015). Interestingly, there is recent evidence that BCRP-mediated breast cancer drug resistance is prevented and reversed by MET (Davies et al. 2017). Subsequently, MET kinetics places it as an attractive tool for studying its effect on BBB BCRP and if it can have a potentially beneficial role in DRE.

In this study, we focus on the role of BCRP in hindering some AEDs e.g., LTG brain entry, and the potential benefits of its inhibition by ko143 and MET on the bioavailability and efficacy of LTG.

## Experimental procedures

### Animals

The present study included 42 male CD1 mice weighing between 20 and 30 g. Animals were housed in the Medical Physiology department animal house, Alexandria Faculty of Medicine, Egypt. They were kept in separate cages and were maintained at a temperature of 23 °C -27 °C, and a 12/12-h light/dark cycle with ad libitum access to food and water. Animals were acclimatized to housing conditions for 1 week before starting the study. All methods were carried out in accordance with ARRIVE guidelines and were approved by the Alexandria Faculty of Medicine Ethics Committee (Ethics approval number: 0305489). Mice were allocated to different groups randomly. Seizure assessment was conducted blindly by personnel who didn’t know the allocated groups or treatments received.

### Drugs and chemicals

Pentylenetetrazole (PTZ), lamotrigine (LTG), and Ko143 were purchased from (Sigma-Aldrich, Saint Louis, USA). Acetonitrile, methanol, and KH_2_PO_4_ buffer used in the chromatographic analysis were purchased from (Fisher Scientific Ltd- Loughborough—UK).

### Experimental grouping

Mice were separated into six equal groups (n = 7/group). G1:Normal control (Vehicle-treated healthy mice): phosphate buffered saline (PBS 0.1 ml/10 g B.W.) was injected intraperitoneally (i.p.) G2:Lamotrigine-treated healthy mice (LTG): mice received LTG (20 mg/kg) i.p., G3: Acute seizures (A.S) mice (PTZ): mice received PTZ (50 mg/kg) i.p., G4: Lamotrigine-treated A.S mice (LTG + PTZ): mice received LTG (20 mg/kg) + PTZ (50 mg/kg) i.p. (Getova and Mihaylova 2011), G5: Ko143 + LTG treated A.S mice (Ko143 + LTG + PTZ): mice received Ko143 (15 mg/kg) i.p. one hour before injection of LTG + PTZ (Wanek et al. 2011); Allen et al. 2002), G6: Metformin + LTG treated A.S mice (MET + LTG + PTZ): mice received metformin (200 mg/kg) i.p. one hour before injection of LTG + PTZ (Hussein et al. 2019). All drugs and chemicals were given in a single dose. In groups 4,5 and 6, PTZ was given immediately after LTG (Getova and Mihaylova 2011).

### Seizure assessment

For 1 h after PTZ injection, mice were observed for seizure assessment (Getova and Mihaylova 2011) by recording seizure latency, frequency, and severity using Ferraro scale (Table 1) (Ferraro et al. 1999). Mice with no seizures during the post-injection observation hour are considered to have seizure latency = 60 min, frequency = 0/min, and severity = 0. Following seizure assessment, the blood samples were collected under isoflurane inhalational anaesthesia. Immediately afterward, mice were decapitated, and brains were divided into two hemispheres, one hemisphere for BCRP expression analysis and the other for LTG brain level assessment. For serum separation, blood samples were left to clot and centrifuged at 5000 xg for five minutes. Serum and brain samples were promptly frozen at -80 °C until analyzed by HPLC and western blot.

**Table 1:** Ferraro scale

Stage 0	No behavioural change
Stage 1	Hypoactivity and immobility
Stage 2	≥ two isolated, myoclonic jerks;
Stage 3	Generalized clonic convulsions with preservation of righting reflex
Stage 4	Generalized clonic or tonic–clonic convulsions with loss of righting reflex

### Measurement of serum and brain LTG by HPLC

#### Standard solution preparation

An external standard stock solution of LTG was prepared by dissolving 10 mg LTG powder in 1 ml of (0.5 ml methanol + 0.5 ml acetonitrile) mixture; then the following concentrations were prepared for the standard curve: 0.1ug/ml, 1ug/ml, 10ug/ml and 50ug/ml(Castel-Branco et al. 2001).

#### Serum and brain samples extraction

**Serum sample** extraction was done by adding 50 μl of each sample to 140 μl of methanol (1% acetic acid). Five-minute vortexing and two-minute centrifugation at 12,000 xg were carried out for protein precipitation. Thereafter, fixed volumes of supernatants were transferred into HPLC sample vials for injection (Jin et al. 2019). **For brain tissue**, each half brain was homogenized at first with phosphate buffer and the supernatant was separated, then sample extraction was performed by the same protein precipitation method of serum samples (Castel-Branco et al. 2001).

#### HPLC Condition

HPLC system was Agilent 1260 Infinity Quaternary LC (USA) with a UV–Vis Detector (USA). Zorbax C18 column (150 × 4.6 mm i.d.) was applied as stationary phase. The software used was Agilent Lab Advisor (USA). A mixture of KH_2_PO_4_ (50 mM) and methanol (61:39) was used for separation at 1.0 ml/min. The column temperature was 37 °C. LTG was monitored at 210 nm for 10 min, and LTG retention time was 6.10 min (Jin et al. 2019).

#### Western blot analysis of BCRP

Radioimmunoprecipitation assay (RIPA) lysis buffer was used for brain homogenization. The supernatant’s protein content was quantified by BioMed protein assay. Total protein in similar amounts was separated by sodium dodecyl sulfate–polyacrylamide gel (SDS-PAGE) and transferred to nitrocellulose membranes (Thermo Scientific, USA). The target protein was probed with primary antibodies against BCRP (#4477, Cell Signaling Technology, USA, 1:1000). Meanwhile, β- actin (#4970, Cell Signaling Technology, USA, 1:1000) was used as a loading control. Goat anti-rabbit alkaline phosphatase-conjugated secondary antibody (#7054, Cell Signaling Technology, USA, 1:2000) was applied, followed by protein visualization with nitro blue tetrazolium/5-bromo-4-chloro-3-indolyl-phosphate (NBT/BCIP) solution (Thermo Scientific, USA). The blots were analyzed with Image Studio Lite Software (LI-COR Biotechnology, Lincoln, NE, USA). The expressed protein was normalized to β- actin and expressed as a fold change (Kammala et al. 2022).

#### Statistical method

Data were analyzed using SPSS software, version 20.0. (NY-IBM). The normality of distribution was tested by Kolmogorov–Smirnov test. Parametric data are expressed as mean ± standard deviation (S.D), Analysis of variance (ANOVA) with Tukey’s tests were used for statistical analysis. Nonparametric data are expressed as median and interquartile range (IQR), Kruskal Wallis test with Dunn's for multiple comparisons test were used for statistical analysis. Correlation studies were performed using Pearson or Spearman coefficients according to variable type and level of distribution. P value ≤ 0.05 was considered significant.

## Results

### Seizure assessment

#### Seizure latency

**Fig. 1:**
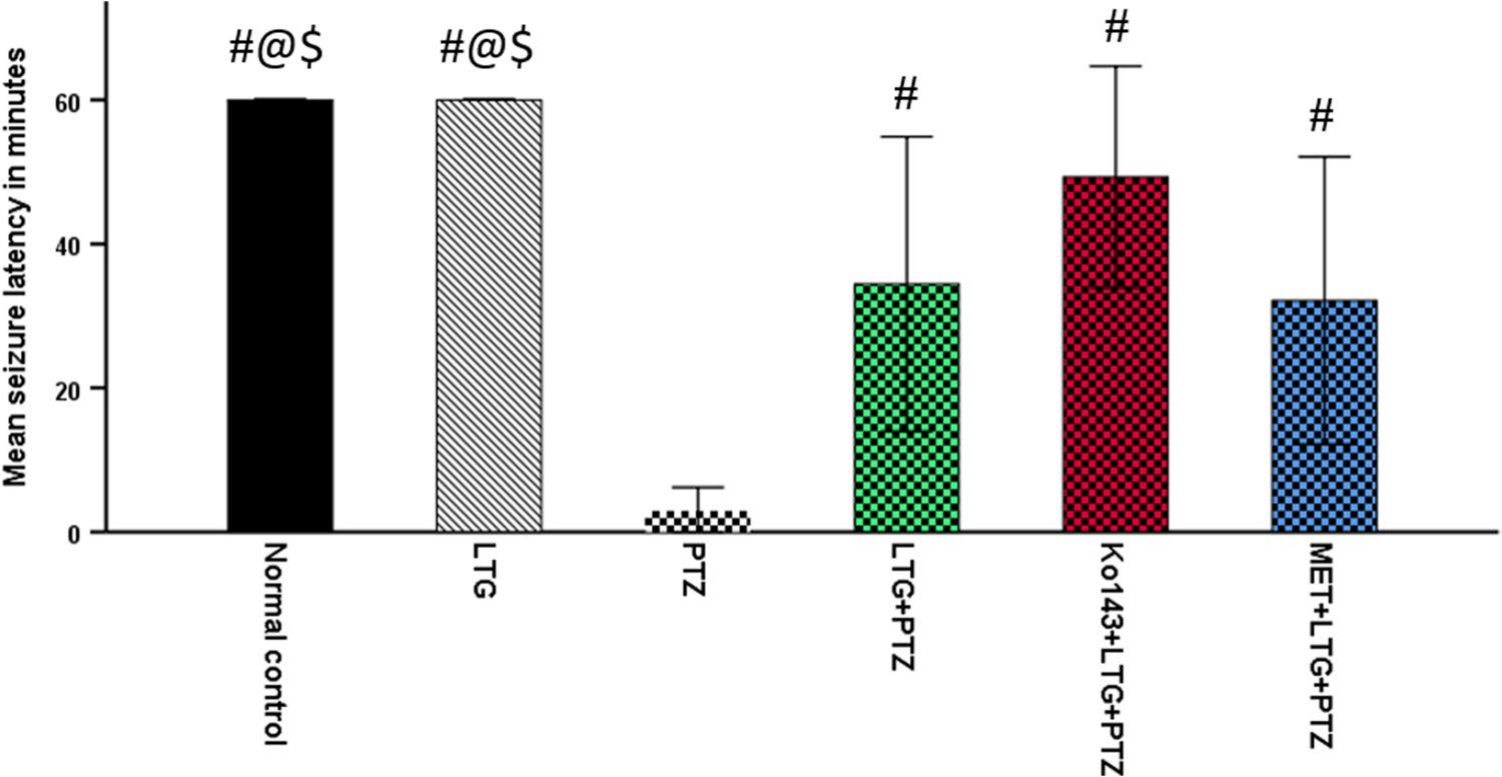
Seizure latency in minutes. Data expressed as mean ± SD (n = 7). One-way ANOVA was performed, pairwise comparison bet. each  2 groups was done using Tukey Test. Significance was denoted by p ≤ 0.05. LTG = lamotrigine, PTZ = Pentylenetetrazole, MET = metformin. #: Significant difference as compared to PTZ group, @: Significant difference as compared to LTG + PTZ group, $: Significant difference as compared to MET + LTG + PTZ group

No seizures were shown in both normal control and LTG groups till the end of 60 min post-injection. In PTZ group, the mean seizure latency was 3 min, which is significantly lower than the other three A.S groups. Among the four A.S groups, the highest mean seizure latency was shown in Ko143 + LTG + PTZ group and was equal to 55 min (Fig. 1).

#### Seizure frequency per minute

**Fig. 2:**
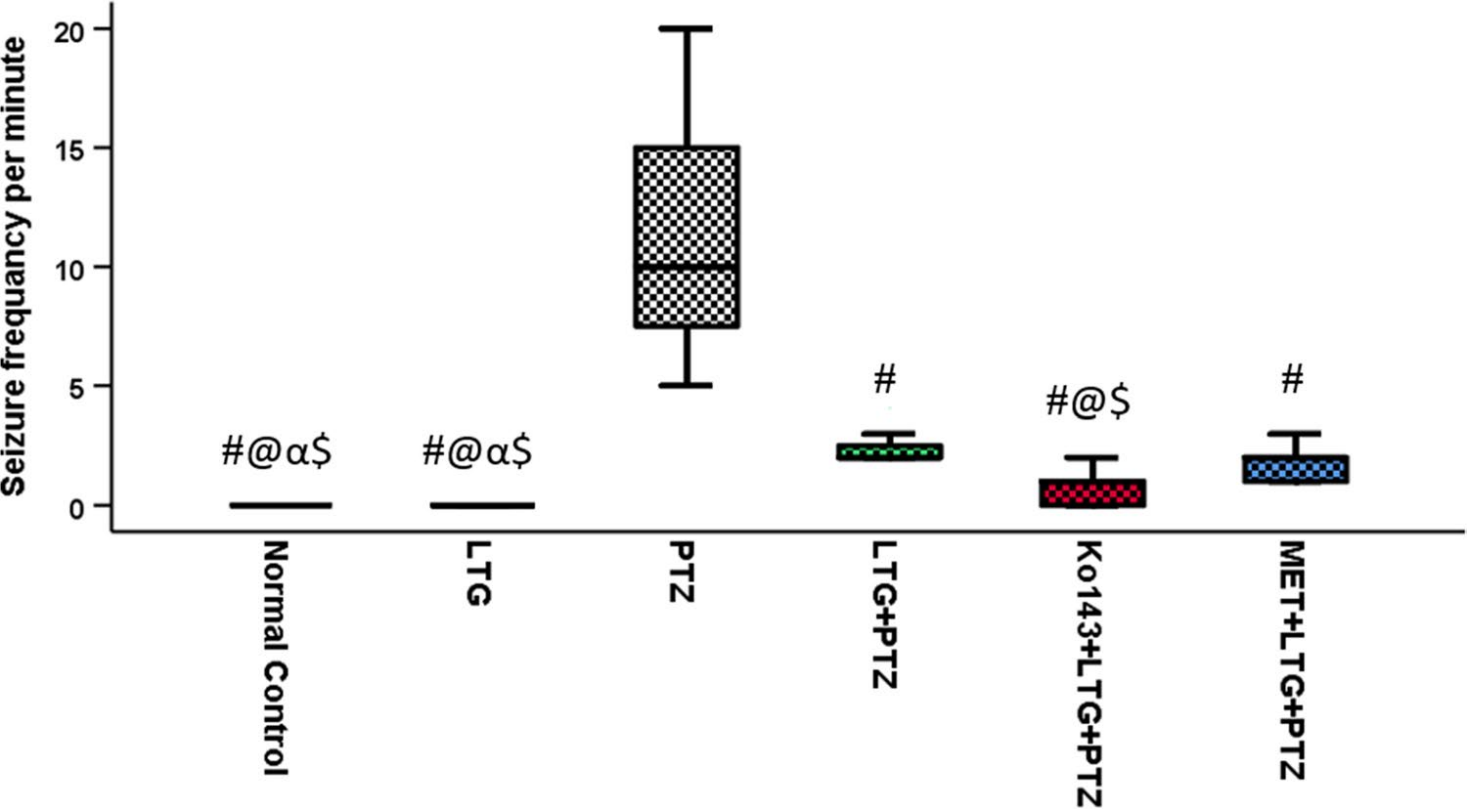
Seizure frequency per minute. Data expressed as median ± IQR (n = 7). Kruskal Wallis test was performed, pairwise comparison bet. each 2 groups was done using Dunn's for multiple comparisons test. Significance was denoted by p ≤ 0.05. LTG = lamotrigine, PTZ = Pentylenetetrazole, MET = metformin. #: Significant difference as compared to PTZ group, @: Significant difference as compared to LTG + PTZ group, α: Significant difference as compared to Ko143 + LTG + PTZ group, $: Significant difference as compared to MET + LTG + PTZ group

In normal control and LTG groups, all mice had no seizures. The highest median value for seizure frequency was seen in PTZ group. LTG + PTZ group has a significantly lower median seizure frequency compared to PTZ group. In Ko143 + LTG + PTZ group, three mice had no seizures and a significant decrease in median seizure frequency compared to PTZ and LTG + PTZ, and MET + LTG + PTZ groups. Median seizure frequency in MET + LTG + PTZ group was equal to LTG + PTZ group and it showed also a significant reduction compared to PTZ group (Fig. 2).

#### Seizure severity score

**Fig. 3:**
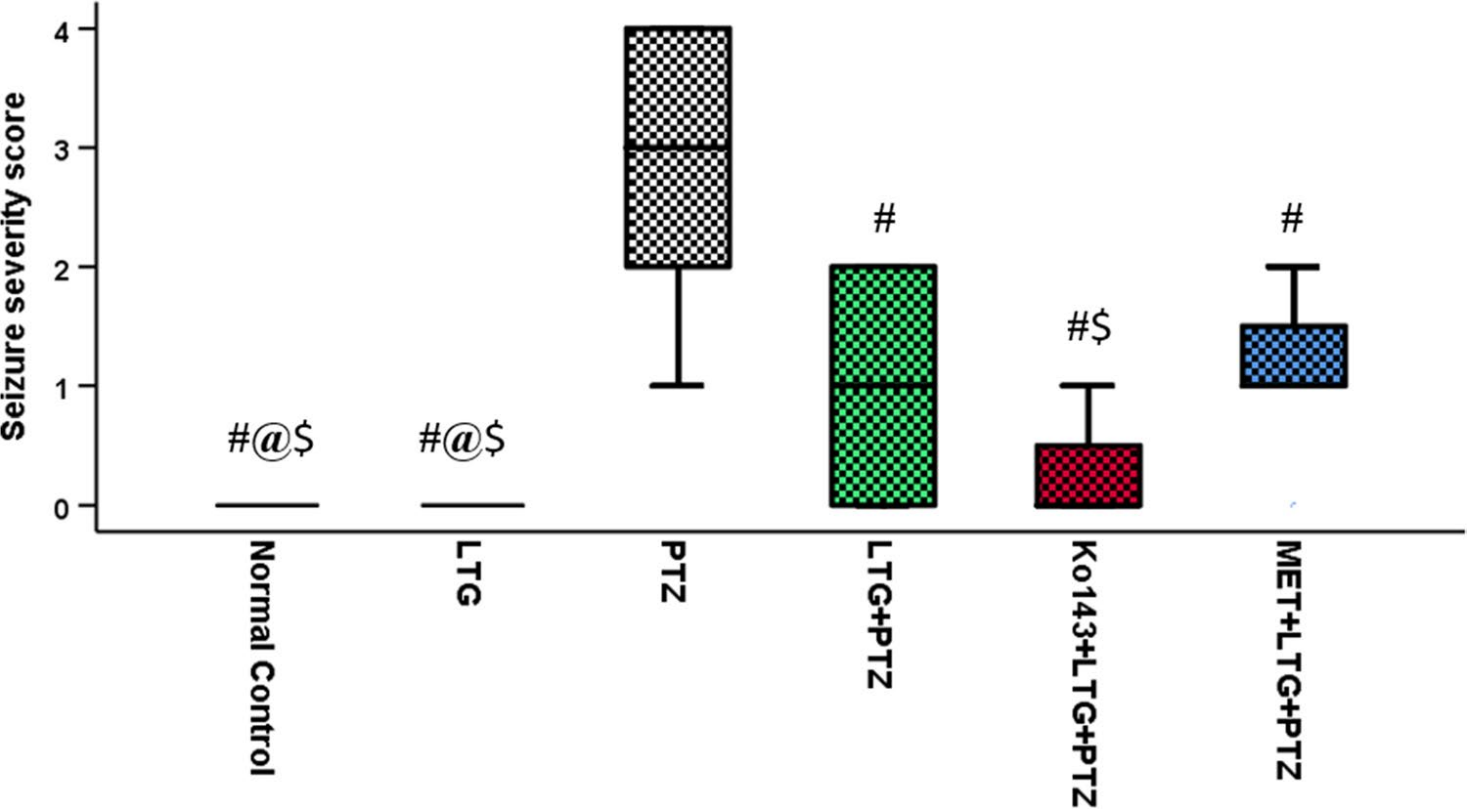
Seizure severity score. Data expressed as median ± IQR (n = 7). Kruskal Wallis test was performed, pairwise comparison bet. each 2 groups was done using Dunn's for multiple comparisons test. Significance was denoted by p ≤ 0.05. LTG = lamotrigine, PTZ = Pentylenetetrazole, MET = metformin. #: Significant difference as compared to PTZ group, @: Significant difference as compared to LTG + PTZ group, $: Significant difference as compared to MET + LTG + PTZ group

The highest severity score was seen in PTZ group with a median value equal to 3. Seizure severity was significantly lower in the three other treated A.S groups with the lowest severity in Ko143 + LTG + PTZ (Fig. 3).

### Serum and brain LTG level (µg/ml)

**Fig. 4:**
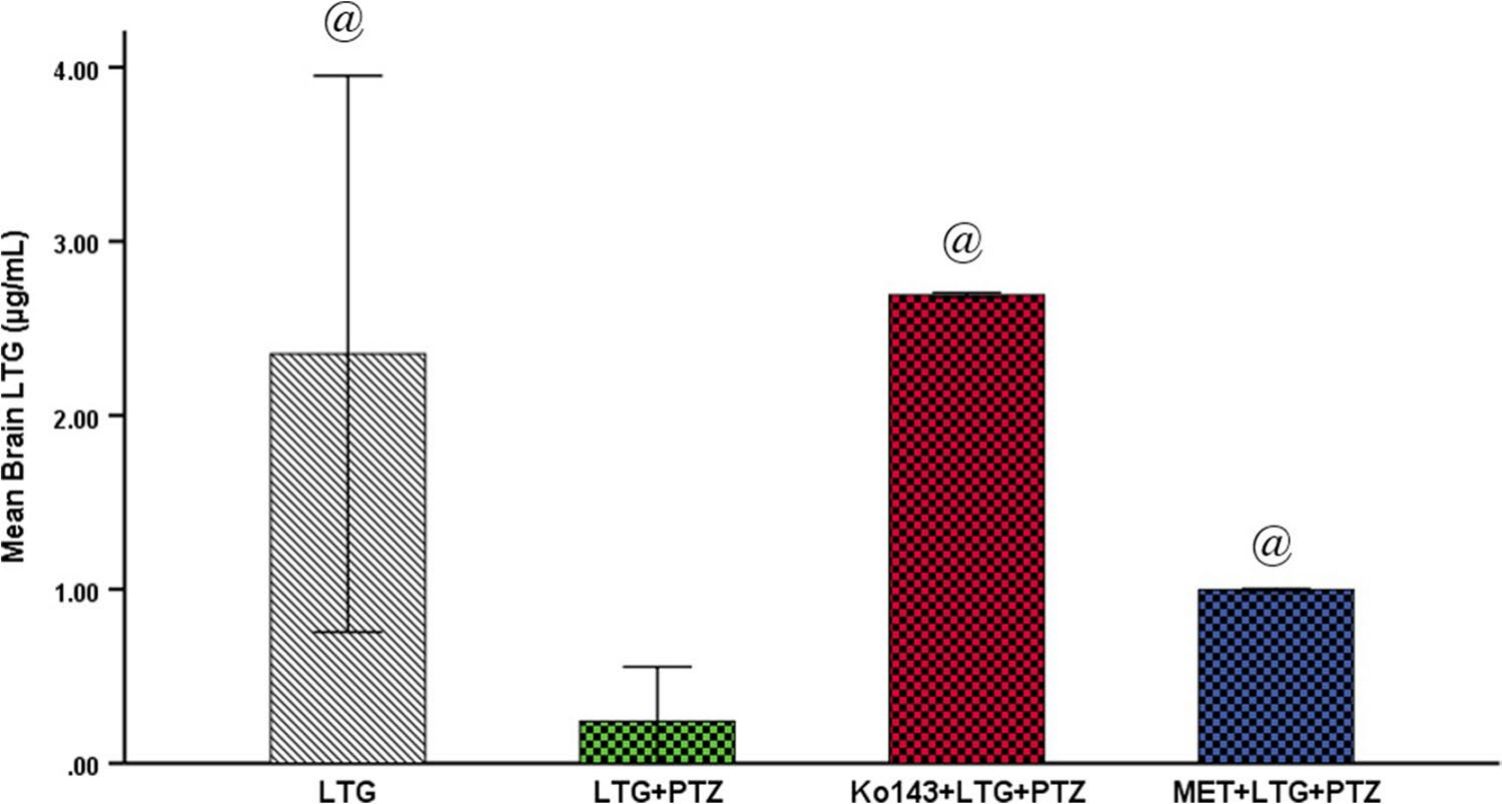
Brain levels of LTG in µg/ml. Data expressed as mean ± SD (n = 7). One-way ANOVA was performed, pairwise comparison bet. each  2 groups was done using Tukey Test. Significance was denoted at p ≤ 0.05. LTG = lamotrigine, PTZ = Pentylenetetrazole, MET = metformin. @: Significant difference as compared to LTG + PTZ group

There was no significant difference in serum LTG among the four LTG-treated groups. Median serum LTG level ranged between (0.13–1.89 µg/ml). While mean brain LTG was significantly lower in LTG + PTZ group compared to LTG-injected healthy mice, Ko143 + LTG + PTZ and MET + LTG + PTZ groups (Fig. 4 and Suppl. Figure 1).

### Brain BCRP level 

**Fig. 5:**
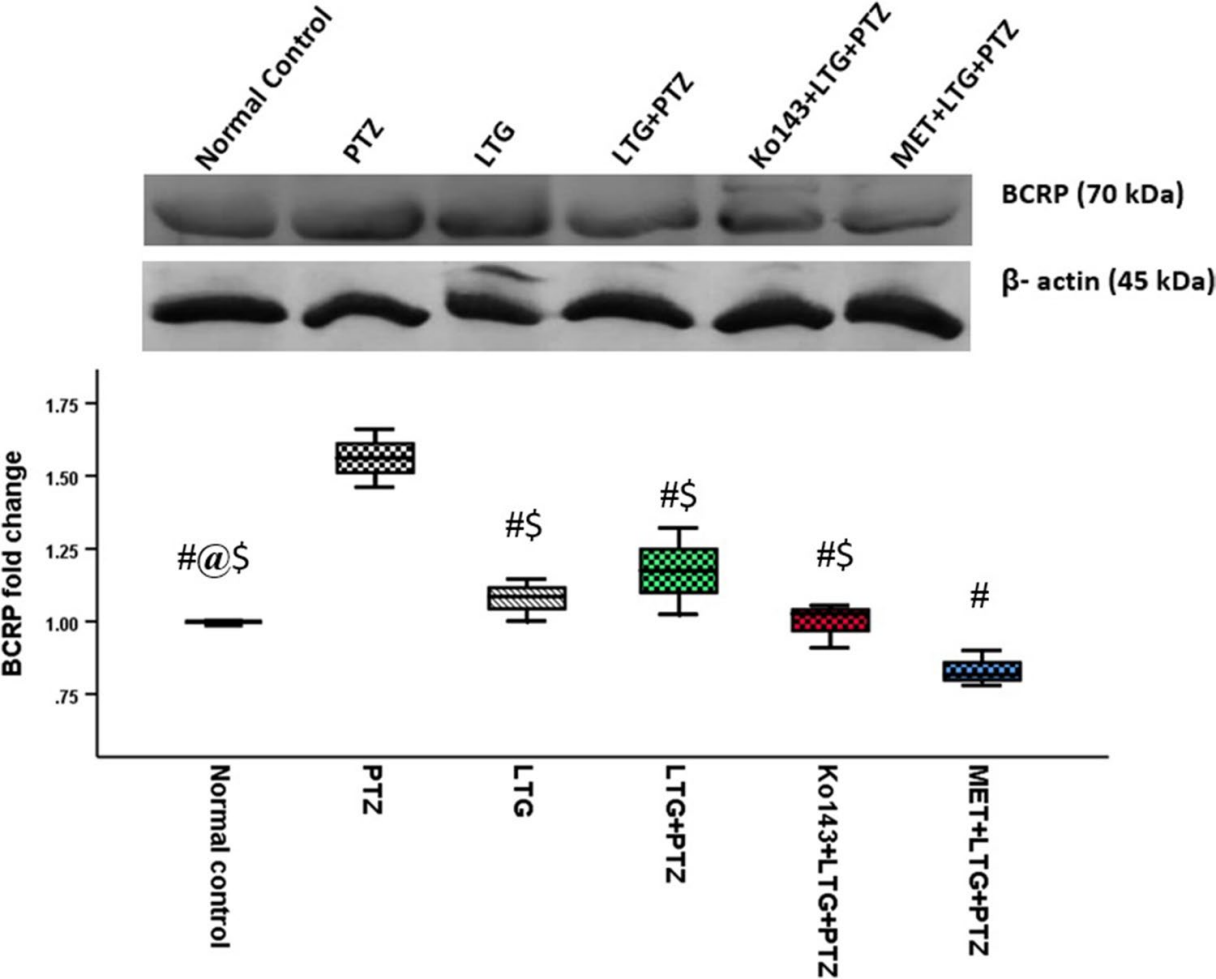
BCRP level in mice brain. Data expressed as median ± IQR (n = 3). Kruskal Wallis test was performed, pairwise comparison bet. each 2 groups was done using Dunn's for multiple comparisons test. Significance was denoted at p ≤ 0.05. PTZ = Pentylenetetrazole, LTG = lamotrigine, MET = metformin. #: Significant difference as compared to PTZ group, @: Significant difference as compared to LTG + PTZ group, $: Significant difference as compared to MET + LTG + PTZ group

Brain BCRP level showed a significant increase in PTZ group compared to other groups. In LTG group, BCRP was lower than LTG + PTZ group. On the other hand, BCRP level was significantly lower in Ko143 and MET injected groups compared to LTG, PTZ, and LTG + PTZ groups. MET-injected group showed the lowest BCRP level (Fig. 5 and Suppl. Figure 2).

### Correlation studies

**Fig. 6:**
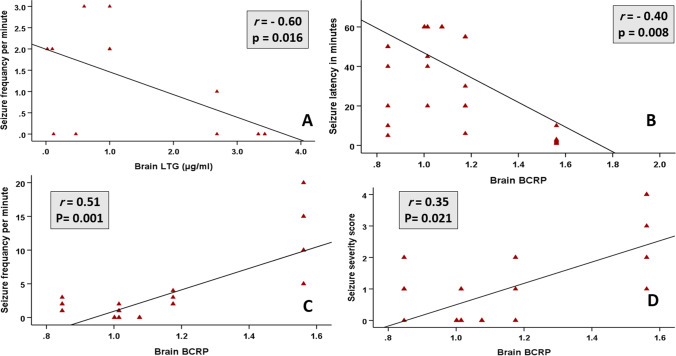
Correlation studies: Scatter diagram showing an inverse correlation between brain LTG and seizure frequency in (**A**) and between brain BCRP and seizure latency in (**B**). A positive correlation between brain BCRP and seizure frequency in (**C**) and seizure severity in (**D**)

No significant correlations were observed between serum LTG and seizure parameters or brain LTG. On the other hand, brain LTG was inversely correlated to seizure frequency. Brain BCRP was correlated positively to both seizure frequency and severity and inversely to seizure latency (Fig. 6).

## Discussion

Resistant epilepsy is a multifactorial and drug-nonspecific clinical problem (Sisodiya 2003). Experimental seizure models provide opportunities to characterize mechanisms of drug resistance that pave the way towards an effective solution to such a problem.

Here, we focused on BCRP's role as a possible mechanism that might contribute to DRE and the potential advantage of its inhibition by Ko143 and MET on improving LTG antiepileptic effect following its acute administration to a mouse model of PTZ-induced acute seizures.

Seizures were induced in the current study by the gamma-aminobutyric acid (GABA)- antagonist, PTZ (Getova and Mihaylova 2011). PTZ is widely used in experimental studies to create a chemically- induced seizure model by suppressing inhibitory synapses and increasing neuronal activity. A single dose of 50 mg/kg was successful in inducing acute generalized seizures in mice. This present study's rationale for seizure induction was adopted from previous several experimental studies that showed this method's success in mice (Suzdak and Jansen 1995; Getova and Mihaylova 2011; Erum et al. 2019).

In the present study, PTZ group compared to other groups experienced the highest seizure frequency and severity and showed a significant increase in brain BCRP expression attributed to seizures. Seizures are proven to be major efflux transporter up-regulators where high glutamate levels produced during seizures activate cytosolic phospholipase A2, resulting in BCRP overexpression (Hartz et al. 2019). This notion is supported by several experimental and clinical studies that discovered a significant correlation between seizure severity and efflux transporters level (Kwan et al. 2002; Hartz et al. 2017; Harby et al. 2020). Interestingly, this increase in brain BCRP expression appeared after 1 h of seizures. Although, Alvariza et al. (Alvariza et al. 2014) study on rat brain showed that efflux transporters expression changes over more than 6 h after AEDs administration, there is strong evidence that some proteins, including efflux transporters, do increase even earlier after exposure to seizures or traumatic brain injury (Bauer et al. 2008; Lin et al. 2012).

There was no significant change in serum LTG among LTG-treated groups. This finding agrees with Clinckers et al. (Clinckers et al. 2008), who reported a lack of significant change in AED serum level by the effect of neither efflux transporters nor their inhibition despite the presence of significant change in AED brain level.

Focusing on LTG + PTZ group, despite the significantly lower brain LTG compared to other groups, seizures were significantly more controlled than in PTZ group. Additionally, the decrease in BCRP expression compared to PTZ group can be explained by the lower seizure severity (Kwan et al. 2002). Besides the role of seizures in inducing BCRP transporter expression, it should be emphasized that the transporter hypothesis of DRE does not only include seizures as a trigger. A growing body of literature suggests that in addition to seizures, AEDs may themselves induce efflux transporters expression (Vázquez and Fagiolino 2022). This evidence appears in the present study through the increased brain BCRP level in LTG group compared to the normal control group.

In the current study administration of Ko143 before LTG + PTZ succeeded in totally aborting seizures in 3 mice out of 7 while in the remaining 4 mice, it increased seizure latency and decreased seizure frequency and severity in comparison to LTG + PTZ group. This evident seizure severity mitigating effect of Ko143 is due to its role in the inhibition of BCRP transporters which was confirmed in our study by the increased brain LTG concentration in Ko143-injected group compared to LTG + PTZ group. Brain BCRP level was lower compared to both PTZ and LTG + PTZ groups. This can be attributable to the evidently better seizure control in Ko143 group. This is consistent with previous studies that have recognized the role of ko143 in increasing the cellular availability of drugs identified as BCRP substrates, hence overcoming their resistance (Yuan et al. 2009; Wanek et al. 2012).

With respect to MET administration before LTG, to our knowledge, this study is considered the first to test the possible chemo-sensitizing effect of MET as an add-on to LTG. MET-injected mice showed significantly better seizure control compared to PTZ group. However, MET seizure mitigating effect was not as much as ko143. This can be attributed to the higher brain LTG level in ko143 group compared to MET group. Ko143's high potency and affinity to BCRP can explain this result where despite the lower amount of BCRP in MET group, the rapid inhibitory effect of ko143 succeeded to elevate brain LTG level more rapidly (Allen et al. 2002; Ni et al. 2010).

Although MET administration did not achieve significantly higher seizure control compared to LTG + PTZ group this cannot rule out MET potential benefit where using different dosing regimens can give a clearer image. MET might require a different dose or a more frequent intake e.g., in chronic epilepsy where daily doses are given (Yang et al. 2017).

Regarding brain LTG level, MET-injected group showed a significant improvement compared to LTG + PTZ group. These results go in line with Hacker et al. (Hacker et al. 2015). and Davies et al. (Davies et al. 2017) who discovered the MET transporter inhibitory effect and its value in the re-sensitization of treatment-resistant breast cancer. Brain BCRP level in MET-injected group showed an interestingly lowest level compared to other groups although it does not have the lowest seizure severity. This interesting finding needs further explanation as it raises the question of whether MET influences BCRP beyond its inhibition. Davies et al. (Davies et al. 2017) answered this question where they found that pretreatment with metformin effectively inhibits the expression of MDR-associated proteins. Besides efflux transporters downregulation, MET has been recently shown to have a neuroprotective and anti-seizure effect with regular intake (Hussein et al. 2019; Sanz et al. 2021).

The significant inverse correlation between brain LTG and seizure frequency in the current study signifies the evidence that seizures through upregulating BCRP hinder LTG brain entry while successful seizure control and BCRP inhibition restore BBB LTG permeability. Additionally, the significant inverse correlation between brain BCRP and seizure latency plus its positive correlation with seizure frequency and severity emphasize that upregulated brain BCRP plays a role in DRE pathogenesis where its higher level can be reflected clinically as more frequent and severe seizures.

The present study results can pave the way for further investigations regarding the clinical targeting of BCRP and the potential usefulness of MET in DRE management.

## Supplementary Information

Below is the link to the electronic supplementary material.

Supplementary file1 (PNG 87 KB) **Suppl.Fig.1**: Typical chromatograms of lamotrigine (LTG), obtained from brain tissues of A: LTG-treated healthy mice, B: LTG-treated acute seizures (A.S) mice, C: Ko143+LTG treated A.S mice, D: Metformin + LTG treated A.S mice. LTG retention time= 6 minutes.

Supplementary file2 (PDF 253 KB) **Suppl.Fig.2**: Original western blots.

## Data Availability

All data is available upon request to the corresponding author.
