# Supplementary figures and images for: Implications of BCRP modulation on PTZ-induced seizures in mice: Role of ko143 and metformin as adjuvants to lamotrigine
Source: Naunyn Schmiedebergs Arch Pharmacol. 2023 Apr 17;396(10):2627–36. doi: 10.1007/s00210-023-02485-7 (PMC10497685; doi:10.1007/s00210-023-02485-7)

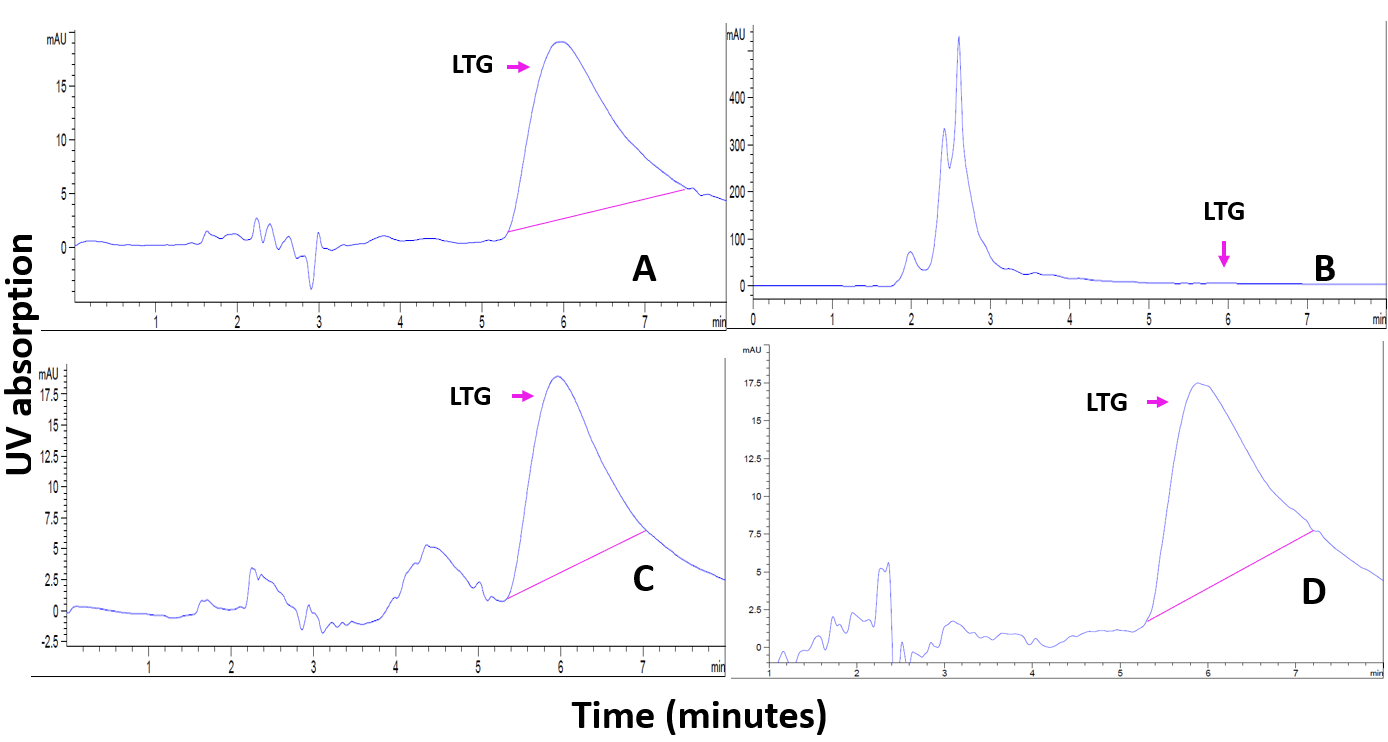

Supplement: Supplementary file 1 — Supplementary file1 (PNG 87 KB) Suppl.Fig.1: Typical chromatograms of lamotrigine (LTG), obtained from brain tissues of A: LTG-treated healthy mice, B: LTG-treated acute seizures (A.S) mice, C: Ko143+LTG treated A.S mice, D: Metformin + LTG treated A.S mice. LTG retention time= 6 minutes. [file 210_2023_2485_MOESM1_ESM.png]
