# Supplementary material for: Implications of BCRP modulation on PTZ-induced seizures in mice: Role of ko143 and metformin as adjuvants to lamotrigine
Source: Naunyn Schmiedebergs Arch Pharmacol. 2023 Apr 17;396(10):2627–36. doi: 10.1007/s00210-023-02485-7 (PMC10497685; doi:10.1007/s00210-023-02485-7)
Supplement: Supplementary file 2 — Supplementary file2 (PDF 253 KB) Suppl.Fig.2: Original western blots. [file 210_2023_2485_MOESM2_ESM.pdf]

Original western blots

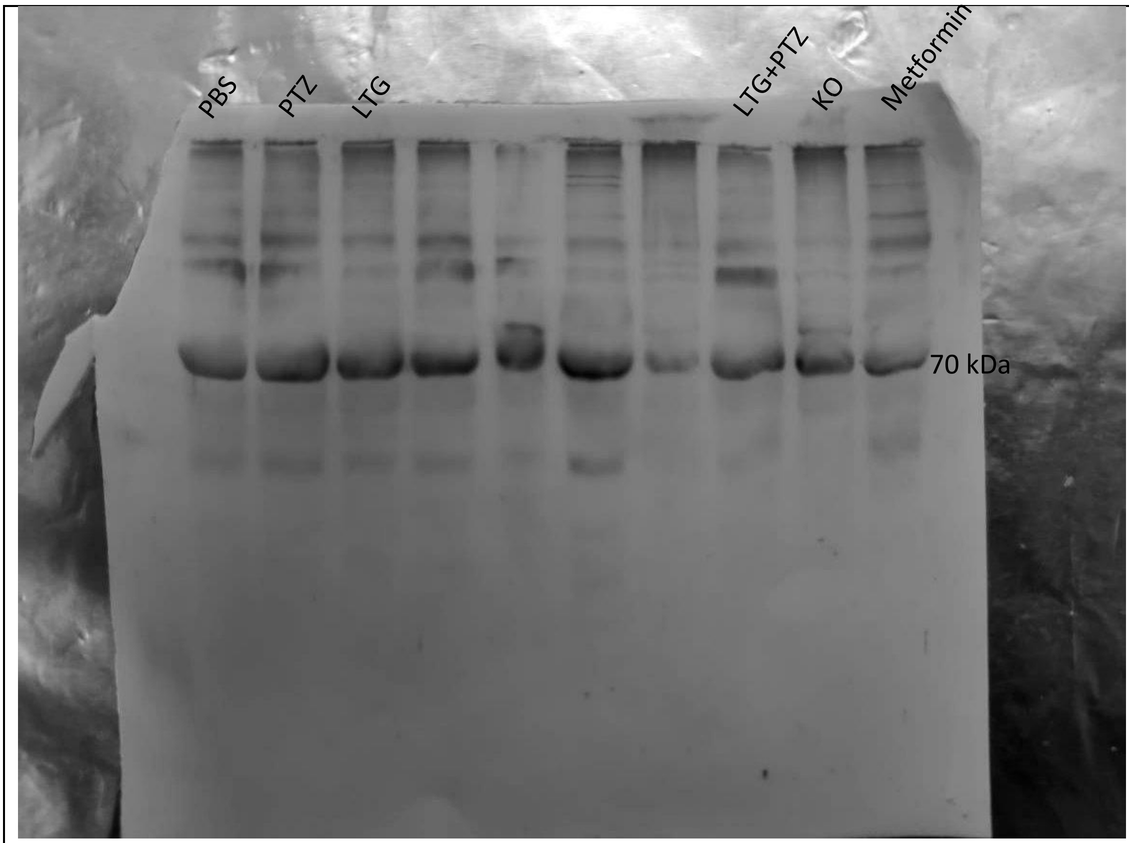	BCRP
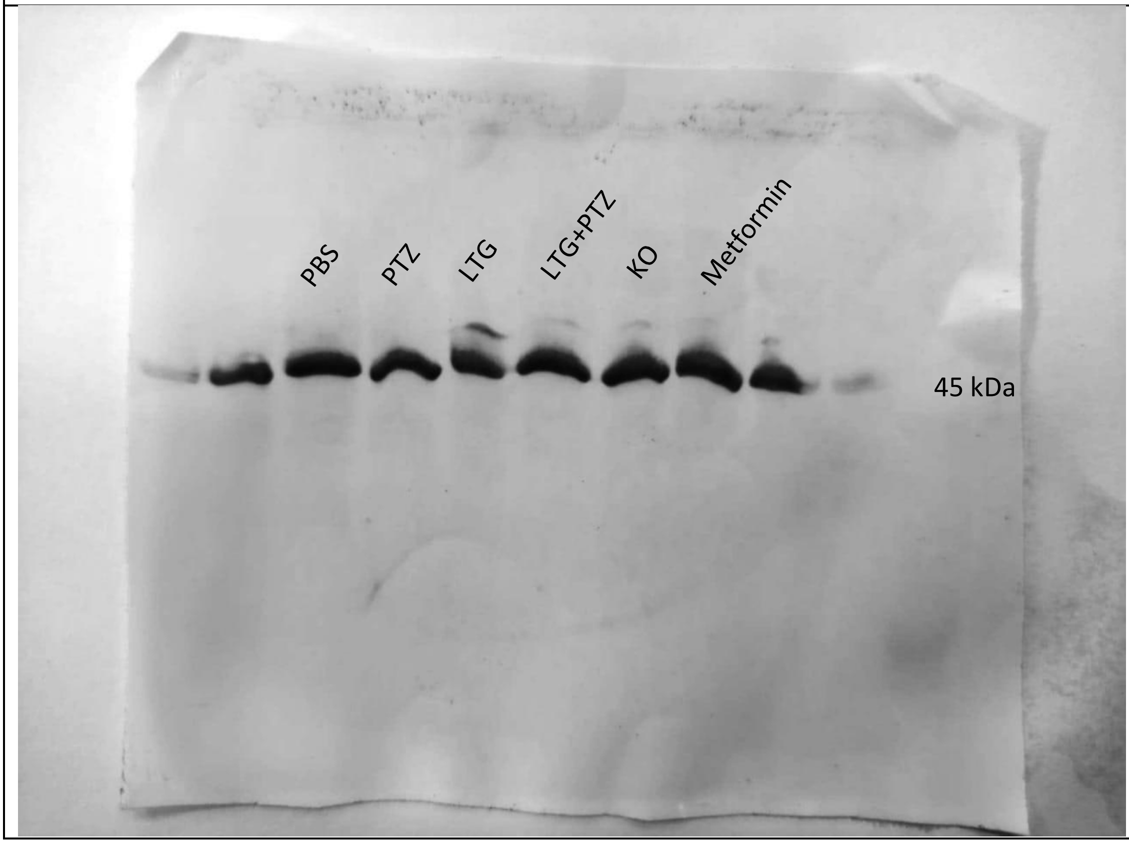	β- actin
